# Genome sequence of *Colletotrichum karsti* isolated from rose leaves exhibiting anthracnose symptoms in Potchefstroom, South Africa

**DOI:** 10.1128/mra.00275-24

**Published:** 2024-09-09

**Authors:** Tawanda E. Maguvu, Adekunle Raimi, Florent P. Trouillas, Rasheed Adeleke, Cornelius C. Bezuidenhout

**Affiliations:** 1 Department of Plant Pathology, University of California, Davis, California, USA; 2 Kearney Agricultural Research and Extension Center, Parlier, California, USA; 3 Unit for Environmental Sciences and Management— Microbiology, North-West University, Potchefstroom, South Africa; University of Strathclyde, Glasgow, United Kingdom

**Keywords:** plant pathogens

## Abstract

We present the genome sequence of *Colletotrichum karsti* isolated from rose leaves exhibiting anthracnose symptoms. The genome was assembled to 53.2 Mbp organized into 753 scaffolds having an N50 of 582,313 kbp and a GC content of 52.5%. The genome had an estimated 99.4% of the core Ascomycota genes.

## ANNOUNCEMENT

*Colletotrichum karsti* infects several host plants, and it is the most common and geographically diverse species in the *Colletotrichum boninense* species complex (1). Despite this, the NCBI database has currently only one publicly available genome for *C. karsti* (strain CkLH20; https://www.ncbi.nlm.nih.gov/datasets/genome/GCF_011947395.1/), thus most of the available genomic information for this species is from a single isolate. To address this gap, we present the genome sequence data of *C. karsti* isolated from leaves of a rose bush (*Rosa hybrida* L; cultivar undetermined) exhibiting typical anthracnose symptoms (location 26.716669 S 27.100000 E). Isolation was done by plating leaf fragments (5 × 5 mm) excised from the margin of lesions on potato dextrose agar following surface sterilization with 1% sodium hypochlorite solution. Genomic DNA was isolated from a hyphal tip-purified single isolate grown in a 250-mL Erlenmeyer flask containing 100 mL of potato dextrose broth (Difco Laboratories), incubated for 7 days at 25°C and 150 rpm. A Quick-DNA Fungal/Bacterial MiniPrep Kit (Zymo Research Group, CA, USA) was used for DNA extraction following the manufacturer’s protocol. Whole genome sequencing was carried out at Novoseq Co. Ltd., Beijing, China. Illumina-based short-read sequencing was carried out using the Novaseq 6000 machine, generating almost equal to 10.6 million reads (two 150-bp reads per spot) with a genome coverage of 30.0×. One microgram of DNA was used as input material, and sequencing libraries were generated using a NEBNext Ultra DNA Library Prep Kit for Illumina (NEB, USA) following the manufacturer’s recommendations. Quality trimming (*Q* > 20) and adapter removal were carried out using Trimmomatic v0.36 (2) with the following parameters: ILLUMINACLIP:TruSeq3-PA.fa:2:30:10 LEADING:3TRAILING:3 SLIDINGWINDOW:10:20 MINLEN:9. Unless otherwise noted, default parameters were used for all software. Assembly of the quality-sequenced reads was performed using SPAdes v3.15.3 (3). Quality assessments of the assembled genomes were performed using QUAST v4.4 (4). The genome of *C. karsti* was assembled to a genome size of 53.2 Mbp organized into 753 scaffolds having an N50 of 582,313 kbp (Table 1). The genome was estimated to have a GC content of 52.5% (Table 1). Assessment of genome integrity by using BUSCO v5.2.2 analysis (5) benchmarking with the fungi_odb10 showed that our genome has 98.8% of the core fungal genes (Table 1).

**TABLE 1:** Quality metrics and annotations of the assembled *Colletotrichum karsti* genome

Assembly size (Mbp)	53.2
Total scaffolds	753
Scaffold N50 (kbp)	582,313
Scaffold L50 count	28
BUSCO estimated completeness	98.8
Coverage (×)	30
GC (%)	52.5
Total predicted genes	14,578
Total predicted proteins	14,560
Total predicted secretome	755
Total predicted effectors	252
Terpene	15
NRPS	9
NRPS-like	10
T1PKS	20
T3PKS	1
Fungal-RiPP-like	14
Isocyanide	1
Total predicted secondary metabolite protein synthesis encoding genes	70
Glycoside hydrolases	424
Auxiliary activities	215
Glycosyltransferases	109
Polysaccharide lyases	49
Carbohydrate-binding modules	42
Carbohydrate esterases	73
Total predicted CAZymes	912

Gene predictions and annotations were performed following the GenSAS v6.0 eukaryotic annotation pipeline (6) as previously described (7) without any modifications. We identified a total of 14,578 genes of which 14,560 were predicted to encode proteins (Table 1). From the predicted proteome, 755 were predicted to be secreted proteins based on the SECRETOOL classical prediction pipeline (8). However, only 252 were predicted to be effector proteins based on EffectorP v3.0 (9) (Table 1). dbCAN3 v3.0.2 (10) predicted a total of 912 CAZymes distributed as shown in Table 1. Fungal antiSMAsh v7.0 (11) predicted a total of 70 secondary metabolite synthesis protein encoding genes (Table 1). Table 1 shows the distribution of the different classes of predicted secondary metabolite synthesis proteins.

**Fig 1:**
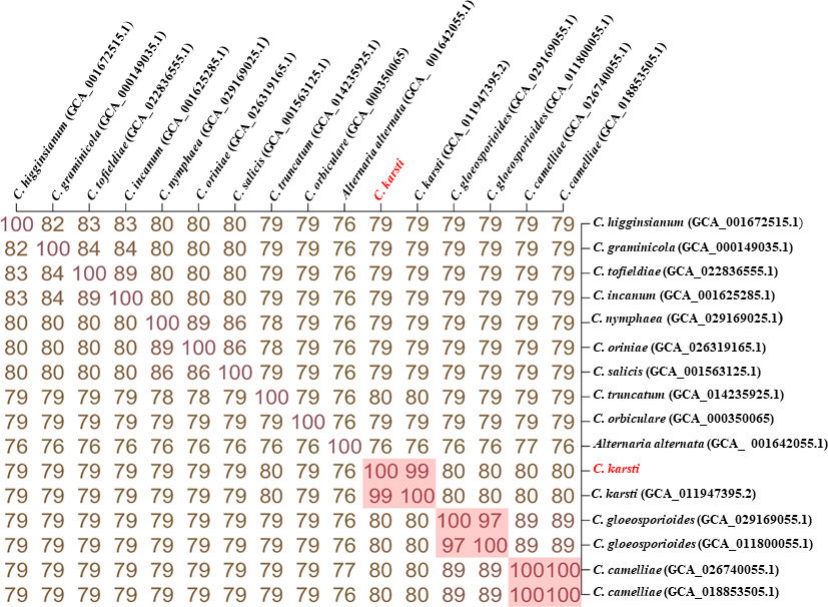
The genome-based distance matrix calculator calculated ANI for *Colletotrichum karsti* and the related genomes. Isolate from this study is highlighted in red.

The genome-based distance matrix calculator [Kostas lab | Genome matrix (gatech.edu)] showed that our isolate shared 99% average nucleotide identity (ANI) with the only available *C. karsti* genome (Fig. 1).

## Data Availability

The whole genome shotgun project (assembly) for *Colletotrichum karsti* isolate TEM has been deposited at DDBJ/ENA/GenBank with the GenBank assembly GCA_038051215.1. The raw sequence data were deposited into the sequence read archive (SRA) under the SRA run accession SRR28292635, and the BioProject PRJNA1086525, with the BioSample SAMN40379275. Data related to the predicted proteins, genes, secretome, effectors, and protein blast have been deposited in the Zenodo repository (https://zenodo.org/uploads/11625333).

## References

[1] Damm U, Cannon PF, Woudenberg JHC, Johnston PR, Weir BS, Tan YP, Shivas RG, Crous PW. 2012. The Colletotrichum boninense species complex. Stud Mycol 73:1–36. doi:10.3114/sim0002 23136457 PMC3458415

[2] Bolger AM, Lohse M, Usadel B. 2014. Trimmomatic: a flexible trimmer for Illumina sequence data. Bioinformatics 30:2114–2120. doi:10.1093/bioinformatics/btu170 24695404 PMC4103590

[3] Bankevich A, Nurk S, Antipov D, Gurevich AA, Dvorkin M, Kulikov AS, Lesin VM, Nikolenko SI, Pham S, Prjibelski AD, Pyshkin AV, Sirotkin AV, Vyahhi N, Tesler G, Alekseyev MA, Pevzner PA. 2012. SPAdes: a new genome assembly algorithm and its applications to single-cell sequencing. J Comput Biol 19:455–477. doi:10.1089/cmb.2012.0021 22506599 PMC3342519

[4] Gurevich A, Saveliev V, Vyahhi N, Tesler G. 2013. QUAST: quality assessment tool for genome assemblies. Bioinformatics 29:1072–1075. doi:10.1093/bioinformatics/btt086 23422339 PMC3624806

[5] Simão FA, Waterhouse RM, Ioannidis P, Kriventseva EV, Zdobnov EM. 2015. BUSCO: assessing genome assembly and annotation completeness with single-copy orthologs. Bioinformatics 31:3210–3212. doi:10.1093/bioinformatics/btv351 26059717

[6] Humann JL, Lee T, Ficklin S, Main D. 2019. Structural and functional annotation of eukaryotic genomes with GenSAS. Methods Mol Biol 1962:29–51. doi:10.1007/978-1-4939-9173-0_3 31020553

[7] Maguvu TE, Travadon R, Cantu D, Trouillas FP. 2023. Whole genome sequencing and analysis of multiple isolates of Ceratocystis destructans, the causal agent of Ceratocystis canker of almond in California. Sci Rep 13:14873. doi:10.1038/s41598-023-41746-6 37684350 PMC10491840

[8] Cortázar AR, Aransay AM, Alfaro M, Oguiza JA, Lavín JL. 2014. SECRETOOL: integrated secretome analysis tool for fungi. Amino Acids 46:471–473. doi:10.1007/s00726-013-1649-z 24370983

[9] Sperschneider J, Dodds PN. 2022. EffectorP 3.0: prediction of apoplastic and cytoplasmic effectors in fungi and oomycetes. Mol Plant Microbe Interact 35:146–156. doi:10.1094/MPMI-08-21-0201-R 34698534

[10] Zheng J, Ge Q, Yan Y, Zhang X, Huang L, Yin Y. 2023. dbCAN3: automated carbohydrate-active enzyme and substrate annotation. Nucleic Acids Res 51:W115–W121. doi:10.1093/nar/gkad328 37125649 PMC10320055

[11] Blin K, Shaw S, Steinke K, Villebro R, Ziemert N, Lee SY, Medema MH, Weber T. 2019. antiSMASH 5.0: updates to the secondary metabolite genome mining pipeline. Nucleic Acids Res 47:W81–W87. doi:10.1093/nar/gkz310 31032519 PMC6602434

